# No evidence for relevant impact of renal replacement therapy on clinical effect of erythromycin in critically ill patients with sepsis

**DOI:** 10.1186/s13054-023-04359-z

**Published:** 2023-02-23

**Authors:** Tom D. Y. Reijnders, Hessel Peters-Sengers, Tom van der Poll

**Affiliations:** 1 Center for Experimental and Molecular Medicine (CEMM), Academic Medical Center, Amsterdam UMC Location University of Amsterdam, Meibergdreef 9, Amsterdam, The Netherlands; 2 Division of Infectious Diseases, Amsterdam UMC Location University of Amsterdam, Meibergdreef 9, Amsterdam, The Netherlands

We appreciate the insightful comments by Prof. Honoré and colleagues regarding our target trial emulation on the effect of erythromycin in critically ill patients with sepsis [1, 2]. Prof. Honoré and colleagues suggest that, in theory, removal of erythromycin from the circulation by renal replacement therapy (RRT) could dilute the potential effect of erythromycin, and that it may therefore be necessary to exclude patients undergoing RRT.

To test their hypothesis, we reanalysed our data after excluding patients that received RRT *within* 72 h of ICU admission (the exposure period) and considered RRT initiated > 72 h *after* admission as a secondary outcome (these patients were not excluded to avoid immortal time bias and selection bias). After excluding 43/235 (18.3%) erythromycin-treated patients and 75/470 (16.0%) control patients, 192 patients remained in the erythromycin group and 395 patients in the control group (Additional file 1: Table S1). When compared with the original study population [2], this subpopulation was similar in terms of baseline demographics, comorbidities and chronic medications, but demonstrated lower disease severity (e.g. APACHE-IV score in the original erythromycin group mean [standard deviation] 90.9 [28.5], new erythromycin group 84.8 [24.1]; in the original control group 85.0 [28.4], new control group 81.4 [26.9]). Within this subpopulation, the number of patients receiving RRT > 72 h after admission was low and not different between groups: 13/192 (6.8%) in the erythromycin group and 26/395 (6.6%) in the control group. Propensity score (PS) matching and (inverse probability of treatment) weighting resulted in well-balanced groups. After matching and weighting, we found no differences in mortality rate up to 90 days: matching HR 0.87 (95% confidence interval [CI] 0.59–1.26), weighting HR 0.91 (95% CI 0.60–1.37) nor in secondary clinical outcomes (aside from a slightly longer ICU length of stay in the erythromycin group in the weighted analysis; *P* = 0.025).

Thus, in our study, RRT does not appear to impact the effect of erythromycin on 90-day mortality to a clinically relevant degree. We would like to emphasize that the pharmacokinetics of macrolide antibiotics in the critically ill are complex, understudied, and affected by other factors, such as accumulation in white blood cells (which are not removed by RRT) [3, 4]. We would very much welcome new studies aimed at better characterizing the pharmacokinetics of macrolide antibiotics in critically ill patients, including the possible elimination by RRT—particularly if these studies also attempt to quantify minimum dosages needed to achieve measurable immunomodulatory effects.

## Supplementary Information

**Additional file 1**. **Supplementary Table 1.** Baseline characteristics and clinical outcomes.

## Data Availability

The datasets used and/or analyzed during the current study are available from the corresponding author on reasonable request.

## Abbreviations

APACHE IV: Acute physiology and chronic health evaluation IV
CI: Confidence interval
HR: Hazard ratio
ICU: Intensive care unit
PS: Propensity score
RRT: Renal replacement therapy
